# Association of Omentin-1 with Oxidative Stress and Clinical Significances in Patients with Breast Cancer

**DOI:** 10.15171/apb.2020.013

**Published:** 2019-12-11

**Authors:** Nahideh Tahmasebpour, Mohammad Ali Hosseinpour Feizi, Nasrin Ziamajidi, Naser Pouladi, Vahid Montazeri, Maryam Farhadian, Roghayeh Abbasalipourkabir

**Affiliations:** ^1^Department of Clinical Biochemistry, School of Medicine, Hamadan University of Medical Sciences, Hamadan, Iran.; ^2^Department of Animal Biology, Faculty of Natural Science, Tabriz University, Tabriz, Iran.; ^3^Department of Biology, Faculty of Basic Sciences, Azarbaijan Shahid Madani University, Tabriz, Iran.; ^4^Department of Thoracic Surgery, Faculty of Medicine, Surgery Ward, Nour-Nejat Hospital, Tabriz University of Medical Sciences, Tabriz, Iran.; ^5^Department of Biostatistics, School of Public Health and Research Center for Health Sciences, Hamadan University of Medical Sciences, Hamadan, Iran.

**Keywords:** Breast neoplasms, Omentin-1, Clinical, Antioxidant, Prognosis, Gene expression

## Abstract

***Purpose:*** Breast cancer (BC) is globally the main reason of cancer-related deaths in women. Omentin-1, an anti-inflammatory and antioxidant adipokine, plays different roles in tumorigenesis and anti-oncogenic pathways. In present study, we investigated the association of omentin-1 with oxidative stress and clinical significances in healthy controls and BC patients to assess the prognostic and diagnostic value of omentin-1 in this cancer.

***Methods:*** This case-control study included 88 BC patients and 86 healthy controls. The serum levels of omentin-1 were assessed by enzyme-linked immunosorbent assays methods. Also, total antioxidant capacity (TAC), total oxidant status (TOS) and malondialdehyde (MDA) serum levels were measured by spectrophotometer. quantitative real-time polymerase chain reaction (qRT-PCR) was applied to the measurement of gene expression of omentin-1.

***Results:*** the serum levels of omentin-1 were significantly lower in the BC patients compared to the healthy controls (*P*<0.001). Moreover, gene expression of omentin-1was significantly downregulated in the BC tissues compared to the adjacent normal tissues (*P*<0.001). Gene expression of omentin-1and its serum levels were significantly higher in grade I compared with grade II and III (*P*=0.001, *P*<0.001, respectively). Additionally, the serum levels of omentin-1 in the p53-positive BC patients were significantly higher than the p53-negative BC patients (*P*=0.001). There was an inverse correlation between the serum levels of MDA and TOS with the serum levels of omentin-1 (r=-0.436, r=-461, respectively).

***Conclusion:*** We conclude that omentin-1 may have a good prognostic and diagnostic roles in the BC patients and decreases oxidative stress in these patients.

## Introduction

Breast cancer (BC), as one of the most prevalent malignancies, is the main cause of cancer-related deaths in females worldwide.^1^ The statistical analysis shows that 76% of common cancers in Iranian women are related to BC. There are about 40 000 patients known with BC in Iran and 7000 new cases are being diagnosed annually.^2^ The average age of BC diagnosis in Iran is about 46 to 49 years which is ten years less than average age in western countries.^3^ Considering the rising rate of BC incidence, early detection and prognosis of this cancer remarkably increases the survival rate of patients.^4^

Several epidemiological studies have demonstrated that weight gain and obesity are significantly related to BC risk, although the molecular mechanism is not understood completely.^5^ Adipose tissue dysfunction in obesity exerts an inflammatory response which may be a crucial cause of BC.^6^ Chronic inflammation causes disturbance in the pro-oxidant/antioxidant balance defined as oxidative stress and formation of reactive oxygen species (ROS) and nitrogen species leading to induction of DNA damage and genetic mutations and inhibition of programmed cell death.^7,8^ Indeed, chronic inflammation and oxidative stress creates a proper microenvironment to promote tumor growth and metastasis in an obese person.^9^

Breast tissue is largely made up of adipose tissue. Therefore, the role of the adipose tissue in initiation and progression of BC has been highlighted in various studies.^10-12^ The adipose tissue, as an important endocrine organ, produces and releases a number of molecules with physiological activity known as adipokines such as leptin, chemerin, resistin and omentin-1.^13^

Omentin-1 (intelectin-1 or intestinal lactoferrin receptor), a peptide with molecular weight of 34 kDa, is an anti-inflammatory and anti-insulin resistance adipokine synthesized abundantly in the visceral adipose tissue.^14^ There are two homologous omentin isoforms known as, omentin 1 and omentin 2, which omentin-1, the principal circulating isoform in plasma, is decreased in obesity and diabetes patients.^15^ According to *in vivo* studies, it has been suggested that omentin-1 is negatively correlated with oxidative stress and is being considered as a positive marker to prevent the damage caused by oxidative stress-induced diabetic nephropathy.^16^

Researchers have shown that omentin-1 has a crucial role in the tumorigenesis and cell differentiation as well as acceleration of apoptosis process in cancer cells.^15,17^ According to *in vitro* studies, it has been showed that omentin-1 has an important role in inducing apoptosis in hepatocellular carcinoma cells^17^ and in another study has been proved that omentin-1 can apply the positive effects in inducing the inhibition of apoptosis, promoting proliferation, increasing secretion of angiogenic cytokine, and stimulate tube formation by human umbilical vein endothelial cells.^18^ Researchers showed that the serum level of omentin-1 is elevated in chronic and acute pancreatitis, while it is decreased in patients with polycystic ovary syndrome and BC.^19-21^ Genetics studies have demonstrated that there are significant relationships between omentin-1 109Asp/Val genotypes and BC risk.^22^

Despite all the available data, there is no evidence for the association of omentin-1 with oxidative stress and clinical significance in patients with BC. Therefore, we evaluated clinical significances and serum markers of oxidative stress and investigated the association of these parameters with the serum levels of omentin-1 and its gene expression in the tissue of women with BC to evaluate the prognostic and diagnostic value of omentin-1 in this cancer.

## Materials and Methods

### Materials

Trichloroacetic acid was obtained from Sigma (St. Louis, MO). Hydrogen peroxide (H2O2), thiobarbituric acid, solvents and other salts were obtained from Merck (Darmstadt, Germany).

### Patients

This case-control study was performed in Nour-Nejat hospital, Tabriz, Iran from April 2018 to September 2018. The BC patients included 88 women with histologically confirmed diagnosis of invasive ductal carcinoma – the most current type of BC^23^ – (age range 32-72 years) who had not received any types of therapy such as surgery with chemotherapy and/or radiation therapy. The healthy controls consisted of 86 women (age range 30-78 years) who had been approved of conducting mammogram screening test over the recent year and not having a family history of any type of cancer. The two groups were matched for age and BMI (body weight divided by height squared (kg/m^2^). After obtaining informed consent, peripheral blood samples were drawn from the BC patients and healthy controls in order to measure the serum omentin-1 level, and also the biochemical and oxidative stress parameters. These samples were immediately centrifuged and their serums were isolated and stored at -20°C until the analysis.

### Assessment of clinical and biochemical parameters

Histopathological data such as stage, grade, size and molecular characteristics of tumor and other clinical features including family cancer history and BMI were collected from medical records and questionnaires. Biochemical parameters including fast blood sugar (FBS), total cholesterol (TC) and triglyceride (TG) were measured with auto-analyzer (Selectra E auto analyzer, Vital Scientific NV, DIERN, Netherland), based on the enzymatic methods in the healthy controls and BC patient groups.

### Assessment of serum omentin-1 levels

Serum concentrations of omentin-1 were assessed by a solid-phase sandwich ELISA kit (ZellBio GmbH, Germany) with Eliza reader (Sunrise, Austria). According to the kit protocol, 174 samples were assessed at the wavelength of 450 nm using a standard curve with a microplate reader. Wavelength correction was performed based on absorbance at 630 nm. This method had a sensitivity of 1 ng/L.

### Real-time qRT-PCR analyses for omentin-1 gene expression

Using Kiazol solution (Kiazist, Iran), total RNA was extracted from the BC tissues and adjacent normal tissues (50 mg) were collected during radical mastectomy. In order to verify the quantity and purity of the of the extracted RNA a NanoDrop spectrophotometer (Thermo Fisher Scientific, USA) was used, and electrophoresis was performed on 1% agarose gel in order to test the extracted RNA integrity. Total RNA (1 µg) was utilized to synthesize cDNA using RevertAid First Strand cDNA Synthesis Kit (GeneAll, South Korea). cDNA was amplified using qRT-PCR Light Cycler^®^ 96 System (Roche Life Science, Deutschland GmbH Sandhofer, Germany) and applying Real Q Plus 2x Master Mix Green (AMPLICON, Denmark). The multistep amplification protocol consisted of one cycle at 95°C for 15 minutes followed by 40 cycles at 95°C for 20 seconds, 58°C for 30 seconds, and then 72°C for 30 seconds. The gene, which was studied, was omentin-1, while the β-actin gene was the internal control.^24^ Primers were designed by using Primer3 software.

Omentin-1;

Forward: 5′-AATAACGAGAGAGCAGCCAAC-3′

Reverse: 5′-CCACTCCAATCAAAACCAGA-3′.

β-actin;

Forward: 5′AGAGCTACGAGCTGCCTGAC-3′

Reverse: 5′AGCACTGTGTTGGCGTACAG-3′.

The 2^-Δct^ method was used to assess omentin-1 relevant gene expression in the BC tissues and adjacent normal tissues.

### Assessment of oxidative stress biomarker

The serum levels of malondialdehyde (MDA) contents were evaluated using a spectrophotometer (UV-1601; Shimadzu, Japan). In other words, the level of thiobarbituric acid reactive substances was measured in each sample.^25^ Determination of total oxidant status (TOS) was carried out based on the oxidation of iron (II) ions to iron (III) ions by serum oxidants in acidic medium.^26^ Iron (III) ions make a colored complex with xylene orange which can be monitored by spectrophotometer to evaluate total amount of oxidant molecules in serum. H_2_O_2_ was used as a calibrator in this assay. Total antioxidant capacity (TAC) was evaluated by Erel method.^27^ This method is based on Fenton reaction in which antioxidants presence in serums suppresses oxidative reactions, leaving the remaining color unchanged. Furthermore, the TAC of the serums can be measured.

### Statistical analysis

The comparison within and between the groups was measured by *t* test and the data were presented as means ± standard deviation (SD). SPSS software (version 21) was used for statistical analysis. The significance level of *P*< 0.05 was considered for all statistical data. Moreover, the receiver operating characteristic (ROC) curves was used to measure the specificity and sensitivity of predicting BC in the serum level in the healthy controls and BC patients, and also gene expression in the BC tissues and adjacent normal tissues by omentin-1. In addition, the sensitivity/specificity at various cutoff values was calculated by using GraphPad Prism 6.

### Clinical and biochemical parameters

The baseline histopathological and the demographic characteristics of the BC patients are listed in Table 1. The median age at diagnosis was 50 years. The tumor was located in the left side in 54 (61.4%) BC patient. Seventy-five percent of patients were in stage I. Table 2 compared the mean of age, BMI and biochemical parameters in the healthy controls and BC patients. No differences in age (*P* = 0.433) and BMI (*P* = 0.952) were found between two groups. Biochemical parameters showed that FBS and TG levels were significantly higher in the BC patients compared to the healthy controls (*P* < 0.001, *P* = 0.030, respectively). Hyperglycemia, or high blood sugar are associated with hyperinsulinemia, as marker of insulin resistance in diabetes.^28^ Hyperinsulinemia leads to increased insulin-like growth factor-1 expression that could be involved in breast carcinogenic processes.^29^ Based on clinic pathological study of BC, researchers have found that elevated serum levels of TG are associated with the increased risk of BC.^30^ In a study by Wei et al, indicate that the serum levels of TG in the BC patients with metastasis were significantly higher than that of patients without lymphatic metastasis.^31^

**Table 1:** Characteristics of the BC patients (n = 88)

**Variable**	**Category**	**No. (%)**
Age (y)	≤ 50	45 (51.1)
	> 50	43 (48.9)
BMI (kg/m^2^)	<25	46 (52.3)
	25-29.99	28 (31.8)
	≥30	14 (15.9)
Pathologic grade	I	21 (23.9)
	II, III	67 (76.1)
Clinical stage	I	66 (75)
	II	14 (15.9)
	III	8 (9.1)
Tumor size (cm)	< 2.5	46 (52.3)
	≥ 2.5	42 (47.7)
Tumor side status	Right side	34 (38.6)
	Left side	54 (61.4)
Lymph node metastasis	N1	64 (72.7)
	N2	13 (14.8)
	N3	11 (12.5)
Estrogen receptor status	Negative	43 (48.9)
	Positive	45 (51.1)
Progesterone receptor status	Negative	58 (65.9)
	Positive	30 (34.1)
Her-2/neu receptor status	Negative	61 (69.3)
	Positive	27 (30.7)
P53	Negative	66 (75)
	Positive	22 (25)

**Table 2:** Comparison of demographics, biochemical parameters and serum markers of oxidative stress in the BC patients and healthy controls

**Variables**	**Healthy controls** **(n=86)**	**BC patients** **(n=88)**	***P*** **value**
Age (y)	48.93 ± 9.16	50.05 ± 9.73	0.433
BMI (kg/m^2^)	24.85 ± 3.72	24.88±4.14	0.952
FBS (mg/dL)	83.45 ± 6.21	98.73 ± 17.75	<0.001^*^
TG (mg/dL)	131.90 ± 37.02	14470±40.20	0.030^*^
TC (mg/dL)	177.36 ± 30.94	181.45 ± 24.51	0.336
MDA (μmol/mL)	3.97 ± 0.52	6.07± 1.30	<0.001^*^
TOS (μmol/mL)	3.50 ± 0.68	6.48 ± 1.22	<0.001^*^
TAC (μmol/mL)	860.94 ± 117.80	670.02 ± 115.01	<0.001^*^

Abbreviations: BMI, body mass index; FBS, fast blood sugar; TG, triglyceride; TC, total cholesterol; MDA, malondialdehyde; TOS, total oxidant status; TAC, total antioxidant capacity. *P ≤ 0.05.

There was no significant difference in TC level between the healthy controls and the BC patients. This finding was corresponded to Laisupasin et al,^32^ whereas our results were not in agreement with the previous study reported by Assiri et al, that serum levels of TC was highly significant in the BC patients compared to the healthy controls.^33^ Thus, this incongruity may be due to various factors such as sample size age, menopausal status and environmental factors have major impacts on study findings.

### Serum omentin-1 levels

**Figure 1:**
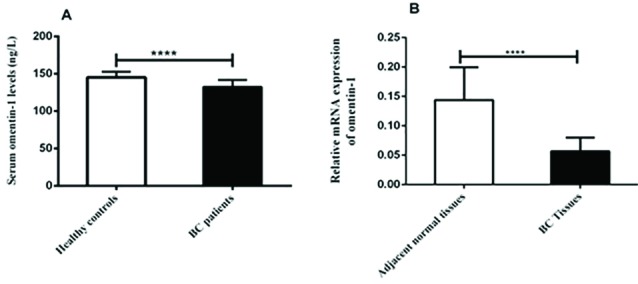
The Serum levels of omentin-1 in the healthy controls and BC patuents (A) and its Relative expression in the adjacent normal tissues and BC patients tissue detected by qRT-PCR (B). ****P value < 0.001.

According to ELISA results, the serum levels of omentin-1 were significantly higher in the healthy controls compared to the BC patients (*P* < 0.001) (Figure 1). The role of omentin-1, an important adipokine produced by adipose tissue, is controversial in various cancers.^19-21^ Studies showed that the serum level of omentin-1 is elevated in chronic and acute pancreatitis, prostate and colorectal cancer, while it is decreased in patients with breast, bladder and renal cell cancers.^21,34,35^ The results of studies suggest that omentin-1 may play different roles in tumorigenesis and anti-oncogenic pathways in cancer.

**Figure 2:**
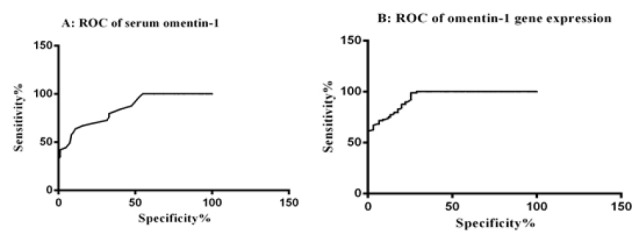
ROC of the serum omentin-1 (A) and its relative expression levels (B) as a diagnostic indicator.

The diagnostic value of the serum levels of omentin-1 were investigated through calculating ROC curve (Figure 2). The best cutoff point for the diagnosis of BC in the serum levels was at 136.5 ng/L. The sensitivity and specificity for the serum levels of omentin-1 with 95% CI (0.791 to 0.902) were 63.64% and 89.02%, respectively. This finding was corresponded to Nourbakhsh et al,^21^ that suggest omentin-1 may be beneficial and invasive markers in the diagnosis of patients with BC.

### Omentin-1 gene expression

**Figure 3:**
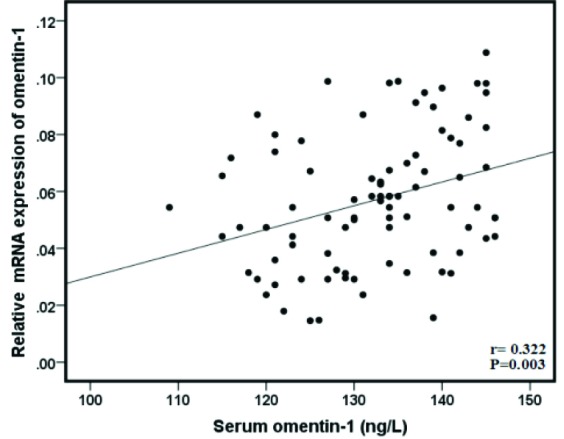
Pearson correlation scatter plot of the serum levels of omentin-1 and its relative expression levels in BC patients which showed a significant positive correlation (Correlation is significant at 0.01 levels).

Gene expression of omentin-1 was evaluated using qRT-PCR. The results showed that gene expression of omentin-1was significantly downregulated in the BC tissues compared to the adjacent normal tissues (*P* < 0.001) (Figure 1). Despite several studies on the serum level of omentin-1 in BC patients, the level of its gene expression in these patients is unclear. In present study, results indicated there was a significant correlation between gene expression of omentin-1and its serum levels in the BC patients (r = 0.322, *P* = 0.003) (Figure 3).

The results of the analysis of ROC curved for gene expression of omentin-1, showed that the AUC and confidence interval (CI) for omentin-1 gene expression were as follows: 0.940 (0.910-0.970) with 96.86% sensitivity and 73.33% specificity. The results of calculating ROC curve showed that gene expression of omentin-1 may have a good diagnostic role in the BC patients.

### Oxidative stress biomarker

Oxidative stress markers in the BC patients and healthy controls are shown in Table 1. The serum levels of MDA as a marker of oxidative stress-induced lipid peroxidation, were significantly lower in the healthy controls compared to the BC patients (*P* < 0.001). Moreover, compared with the BC patients, a notable decrease in the serum levels of TOS and increase in serum levels of TAC are shown in the healthy controls (*P* < 0.001). The higher level of serum MDA in BC patients has been reported in another study.^36^ Other oxidative stress parameters including TAC and TOS have been studied in BC. In a study by Feng et al, the serum level of TOS was higher in the BC group than the healthy group.^37^ There are a bunch of studies that reveal the importance of reactive oxygen species in the initiation and progression of BC.^36^ In study by Ching et al highlighted the correlation between the higher level of total antioxidant status and the lower BC risk.^38^

### Association of omentin-1 gene expression and its serum levels with clinical and biochemical parameters in BC patients

As shown in Table 3, the results demonstrated that the serum levels of omentin-1were significantly higher in BC patients aged ≤50 years than BC patients aged >50 years (*P* = 0.001). the serum levels of omentin-1 were higher in lean BC patients than overweight/obeseBC patients (*P* < 0.001). The results demonstrated in Table 3 that the serum levels of omentin-1 and its gene expression were inversely significant associated with BMI (r = -0.764, *P* < 0.001; r = -0.220, *P* = 0.040, respectively) BMI is one of the most important clinical significances in the BC patients, and there are conflicting results surrounding the correlation between BMI and BC. While some studies indicate the positive relationship between BMI and BC risk, the others showed an inverse correlation.^39-42^ However, the molecular mechanism of the association between obesity and BC is not completely understood, which could be due to the fact that various factors such as age, menopausal status and environmental factors have major impacts on study findings.^43^

**Table 3:** Association the serum levels omentin-1 and its relative mRNA expression levels in tissues with clinicopathological markers in the BC patients

**Variable**	**Serum Omentin-1 (ng/L)** **Mean ± SD**	***P*** **value**	**Omentin-1 relevant expression (2**^-Δct^**)** **Mean ± SD**	***P*** **value**
Age (y)		0.001^*^		0.902
≤50	135.61 ± 8.14		0.05 ± 0.02	
>50	129.00 ± 8.94		0.05 ± 0.02	
BMI (kg/m^2^)		<0.001^*^		0. 104
<25	138.00 ± 6.33		0.05 ± 0.02	
25-29.99	128.25 ± 5.33		0.05 ± 0.02	
≥30	122.14 ± 9.16		0.04 ± 0.01	
Pathologic grades		<0.0001^*^		0.001^*^
I	139.14 ± 4.85		0.07 ± 0.02	
II, III	130.01 ± 8.94		0.05 ± 0.02	
Clinical stages		0.551		0.846
I	132.70 ± 9.76		0.05 ± 0.02	
II	132.00 ± 10.31		0.05 ± 0.03	
III	129.00 ± 9.22		0.05 ± 0.01	
Tumor size (cm)		0.808		0.563
<2.5	132.47 ± 4.85		0.05 ± 0.02	
≥2.5	132.00 ± 9.36		0.05 ± 0.02	
Tumor side status		0.224		0.963
Right side	133.78 ± 9.54		0.05 ±0.02	
Left side	131.28 ± 8.62		0.05 ± 0.02	
Lymph node metastasis		0.560		0.954
N1	132.53 ± 9.45		0.05 ± 0.02	
N2	133.15 ± 8.95		0.05 ± 0.02	
N3	129.54 ± 6.40		0.05 ± 0.01	
Estrogen receptor status		0.107		0.503
Negative	133.81 ± 8.77		0.05 ± 0.02	
Positive	130.67 ± 9.09		0.05 ± 0.02	
Progesterone receptor status		0.060		0.319
Negative	133.55 ± 8.88		0.05 ± 0.02	
Positive	129.73 ± 8.87		0.06 ± 0.02	
Her-2/neu receptor status		0.281		0.433
Negative	131.50 ± 8.80		0.05 ± 0.02	
Positive	133.85 ± 9.44		0.05 ± 0.02	
p53		0.001^*^		0.678
Negative	130.40 ± 8.78		0.05 ± 0.02	
Positive	137.59 ± 7.59		0.05 ± 0.02	

*P< 0.05.

Additionally, gene expression of omentin-1 and its serum levels were significantly higher in grade I compared to grade II and III (*P* = 0.001, *P* < 0.0001, respectively) as shown in Table 3. Currently, histological grade presents the morphological assessment and the degree of differentiation of the tumor tissue and has been shown to generate significant information related to the clinical behavior of BC that is considered to be the basic parameter for determining the prognosis of BC,^44^ and the reverse relationship observed between them suggests that the serum level of omentin-1 and its gene expression may have prognostic values. Additionally, the serum levels of omentin-1 in the p53-positive BC patients were significantly higher than the p53-negative BC patients (*P* = 0.001). In study by Zhang et al, suggest that omentin-1 increases the stability of p53 protein by reducing deacetylation of p53.^17^ Other variables like stage, tumor size and side, hormone receptors (ER, PR, Her2/neu) and lymph node metastasis were not significantly correlated with gene expression of omentin-1 and its serum levels (Table 3).

### Association of gene expression of omentin-1 and its serum levels with oxidative stress markers

Despite all the available data, there is no evidence for the association of omentin-1 with oxidative stress in BC patients. In current study the results showed that, there was an inverse correlation between the serum levels of MDA and TOS with the serum levelofomentin-1 in the BC patients (r = -0.436, r = -0.461, respectively) (Table 4). The serum level of TAC had positive correlation with the serum level of omentin-1 and its gene expression in the BC patients (r=0.411, *P* < 0.001; r = 0.253, *P* = 0.017 respectively) (Table 4). In vitro studies have suggested that omentin-1 exerts its antioxidant effect via regulating the Nrf2/Keap-1 signaling pathway in glomerular mesangial cells (HBZY-1).^16^ In a study by Yin et al, omentin-1 showed antioxidant effects on MSCs by reducing ROS and protecting the function of mitochondria via PI3K/Akt.^18^ *In vitro* study by Gu et al, suggested that omentin-1 inhibits oxidative stress and decreases apoptosis by stimulating the Akt-eNOS signaling pathway in response to ischemia.^45^

**Table 4:** Association of serum stress oxidative marker and biochemical parameters with relative expression of omentin-1 and its serum levels in BC patients

**Variable**	**Serum Omentin-1(μg/mL)**		**Omentin-1 relevant expression (2**^∆-ct^ **)**	
	**r**	***P*** **value**	**r**	***P*** **value**
FBS (mg/dL)	-0.440	<0.001	-0.127	0.238
TG (mg/dL)	-0.185	0.088	-0. 158	0.140
TC (mg/dL)	-0.130	0.233	0.015	0.0891
MDA (μmol/mL)	-0.436	<0.001*	-0.153	0.158
TOS (μmol/mL)	-0.461	<0.001*	-0.201	0.0.60
TAC (μmol/mL)	0.411	<0.001*	0.253	0.017*

Abbreviations: FBS, fast blood sugar; TG, triglyceride; TC, total cholesterol; MDA, malondialdehyde; TOS, total oxidant status; TAC, total antioxidant capacity. *P ≤ 0.05.

## Conclusion

In conclusion, the present study revealed that gene expression of omentin-1 and its serum levels may have a good diagnostic role in BC patients and they had strong correlation with oxidative stress indices and decreased oxidative stress in these patients. The small sample size lacking follow-up time may be considered as the main limitation of this study which may have affected these results. However, further evaluations in a larger patient population as well as in vitro on cancer cell line studies are needed for determining the tumorigenesis and anti-oncogenic signaling pathways.

## Ethical Issues

The Research Committee of Hamadan University of Medical Sciences approved this study (ethical code: IR.UMSHA.REC.1396.915).

## Conflict of Interest

The authors declare that they have no conflicts of interest.

## Acknowledgments

This study, extracted from a PhD dissertation (Project no. 970128368), was approved and funded by Hamadan University of Medical Sciences, Hamadan, Iran. We would like to thank the patients, staff and nurses in the surgical room and the pathology Department of Tabriz Nournejat hospital who sincerely helped us in this project.
